# Supplementary material for: Acceptability and feasibility of home and hospital follow-up in Burkina Faso and Guinea: A mixed-method study among patients of the COVID-19 Coverage-Africa clinical trial
Source: PLOS Glob Public Health. 2023 Jul 12;3(7):e0001545. doi: 10.1371/journal.pgph.0001545 (PMC10337897; doi:10.1371/journal.pgph.0001545)
Supplement: S1 Table — (DOCX) [file pgph.0001545.s001.docx]

**S1 Table. Participants’ subjective assessment of the appropriateness** **of the different follow-up models of the Coverage-Africa trial, mapped according to the Theoretical Framework of** **Acceptability (Sekhon et al., 2017). 2021-2022.**

| **Themes** | **Hospital-based follow-up** | **Home-based follow-up** | **Phone-call follow-up** |
| --- | --- | --- | --- |
| **Construct 1: Affective attitude** | | | |
| **COVID-19 symptoms** | + Acceptable if moderate/severe symptoms | + Acceptable if no/mild symptoms |  |
| **Comfort** | +/- Perception varied according to accommodation conditions | + More comfortable in their living environment |  |
| **Psychological health** | - Can be stressful as no possibility to have bedside support from the family | + Feeling of not being sick when at home |  |
| **Construct 2: Burden** | | | |
| **Practicality (transportation time)** |  | + No need to go to a health facility (time saved) | + No need to go to a health facility (time saved) |
| **Follow-up assessments by the medical teams** |  | + Not difficult to find a private place to receive the medical team for face-to-face assessments | + Not difficult to talk about health over the phone  + Generally not difficult to find a private place to discuss with the medical team over the phone…  - …except if shared rooms in health facilities  - If phone calls at an unscheduled time: can be disturbing |
| **Relations with family / entourage / neighbors** | + Discretion about the COVID-19 status | - Fear of contaminating family members  - Fear of stigmatization |  |
| **Construct 3: Ethicality** | | | |
| **Care from the medical teams** |  | + Perception of being considered/well taken care of by the medical team | + Perception of being considered/well taken care of by the medical team |
| **Construct 4: Intervention coherence** | | | |
| **Complementarity between follow-up models** |  |  | + Phone calls perceived as a complement to home-based follow-up |
| **Medical teams’ workload** |  |  | + Relief for the medical team when high number of patients to follow |
| **Construct 5: Opportunity costs** | | | |
| **Family commitments** | - Difficulty to manage family life | + Ease in managing family life |  |
| **Construct 6: Perceived effectiveness** | | | |
| **Evolution of COVID-19 symptoms** | + Reassuring that the medical team can act/intervene rapidly in case of aggravation |  | + Reassuring to be able to rapidly join the medical team in case of aggravation |
| **Care from the medical teams** |  | + Personalized counselling as the medical team can see life environment  + Perception of continuity of care | + Perception of continuity of care, provided that phone follow-ups are complementary to home visits  - Medical team sometimes unavailable to answer calls |
| **Construct 7: Self-efficacy** | | | |
| **Treatment intake** |  | + No specific difficulties |  |

+: positive aspects; -: negative aspects
