# Supplementary material for: Acceptability and feasibility of home and hospital follow-up in Burkina Faso and Guinea: A mixed-method study among patients of the COVID-19 Coverage-Africa clinical trial
Source: PLOS Glob Public Health. 2023 Jul 12;3(7):e0001545. doi: 10.1371/journal.pgph.0001545 (PMC10337897; doi:10.1371/journal.pgph.0001545)
Supplement: Attachment — Submitted filename: Response to Reviewers.docx [file pgph.0001545.s002.docx]

**To the editors of the Journal of the PLOS Global Public Health**

Dear Editors,

We thank the reviewers for their helpful comments, which we believe have further strengthened our manuscript. We have addressed the queries in a point-by-point response document and have modified the text of our manuscript accordingly. Changes to the manuscript are in "track changes" mode in the manuscript document, and are described below in red.

***Journal Requirements***

1. Please send a completed 'Competing Interests' statement, including any COIs declared by your co-authors. If you have no competing interests to declare, please state "The authors have declared that no competing interests exist". Otherwise please declare all competing interests beginning with the statement "I have read the journal's policy and the authors of this manuscript have the following competing interests:"

**Response.** We confirm that no author has competing interest to declare, and we have clearly stated that “*The authors have declared that no competing interests exist*” in the submission form.

2. In the online submission form, you indicated that "*Study data will not be publicly available. Data can be made available by the sponsor (ANRS) to any researcher interested. Deidentified participant data can be made available and shared under a data transfer agreement. Requests for access to the Coverage Africa study data should be sent to the corresponding author*". All PLOS journals now require all data underlying the findings described in their manuscript to be freely available to other researchers, either 1. In a public repository, 2. Within the manuscript itself, or 3. Uploaded as supplementary information.

This policy applies to all data except where public deposition would breach compliance with the protocol approved by your research ethics board. If your data cannot be made publicly available for ethical or legal reasons (e.g., public availability would compromise patient privacy), please explain your reasons by return email and your exemption request will be escalated to the editor for approval. Your exemption request will be handled independently and will not hold up the peer review process, but will need to be resolved should your manuscript be accepted for publication. One of the Editorial team will then be in touch if there are any issues.

**Response.** The ANRS COV33 COVERAGE Africa study is sponsored by the ANRS MIE (French Agency for Research on AIDS and viral hepatitis / Emerging Epidemic Diseases). We are therefore obliged to abide by French and European legal restrictions such as French law no. 78-17 of January 6, 1978 relating to data processing, files and freedoms usualy called "Loi informatique et libertés" and European law "GDPR" General Data Protection Regulation that govern data sharing and stipulate that data cannot be shared in open access to an undeclared thrid party if study participants have not been informed and did not give their consent for it. For the Coverage Africa trial, consent form indicated that "Your information, called "data", will be encrypted and stored in a database located at the PACCI Research Program in Abidjan and at MEREVA in Bordeaux, France. Only authorized study personnel will have access to the database. [...] Your data may be transferred to French, national or foreign health authorities (drug agency, etc.), to other private or public national or international research teams to be used for other research purposes (new statistical or qualitative analyses). Any transfer will be carried out according to guarantees ensuring their confidentiality and provided for in a contract/agreement on sharing between Inserm-ANRS and the recipient(s) of the data."

Thus, persons wishing to have access to the interviews or questionnaire data may submit a request to the study sponsor (ANRS, [clinique@anrs.fr](mailto:clinique@anrs.fr)), who will take steps to assess whether the request can be fulfilled. That said, we would like to emphasize that all quantitative and qualitative (verbatim extracts) data allowing to answer our study questions are presented in the paper.

***Reviewer's Comments to the Author***

**Reviewer #1:**

Dear authors,

Firstly, I congratulate the authors for the dedication and focus to fortify the science in public health thematic area. The manuscript is important to the public health and provides high importance to better understanding the acceptability and feasibility of different modalities of follow-up among patients with mild to moderate symptoms enrolled in COVID-19 clinical trials.

I strongly suggest to the authors to follow the PLOS Global Public Health’s submission guidelines. There is no line numbering which difficulted the review of the manuscript.

**Response.** We are very sorry to have missed important submission recommendations from PLOS Global Public Health, we have added line numbers in the re-submitted version of the manuscript.

I suggest reading the manuscript carefully, since there are many little mistakes that could be solved easily by the authors.

The manuscript requires professional editing in the English language to make it easy to read and understand.

**Response.** We thank the reviewer for their suggestion. The manuscript has been reviewed to correct mistakes. The corrections are highlighted in the manuscript with tracked changes. Of note, the translated quotes reflect as much as possible the original language level and grammar, which may entail some mistakes.

I suggest to the authors The Good Reporting of a Mixed Methods Study (GRAMMS) criteria to assess reporting quality of Mixed Method studies. It is available from O'Cathain A, Murphy E, Nicholl J. The quality of mixed methods studies in health services research. J Health Serv Res Policy. 2008;13(2):92-98. And <https://www.equator-network.org/reporting-guidelines/the-quality-of-mixed-methods-studies-in-health-services-research/>

**Response.** We thank the editor for their suggestion. We have checked our manuscript according to the GRAMMS criteria as explained below:

- (1) Describe the justification for using a mixed methods approach to the research question: We used a mixed-methods approach in order to provide a comprehensive assessment of the experiences and perceptions of the study participants. The quantitative data aimed to measure in a standardised way the level of participants’ satisfaction and agreement regarding specific statements in a way that would facilitate comparisons between the different follow-up models and between countries. We also originally planned to identify factors (e.g., type of housing, level of education) associated with patients’ satisfaction, which was not possible because of the lack of variability in responses and the very low % of participants dissatisfied. Qualitative data aimed to explore more deeply participants’ individual experiences, views and feelings, and therefore enrich the quantitative findings with subjective and contextual factors.

In order to clarify this in the manuscript, we have added the following paragraph (l.143-149): “*The quantitative component aimed at measuring, in an exhaustive and standardised way, the level of patients’ satisfaction and agreement regarding specific statements, and facilitating comparisons between the different follow-up models and between countries. The qualitative component aimed at exploring in depth participants' individual experiences, views, and feelings about these modalities of follow-up. Combining both quantitative and qualitative data aimed at providing a comprehensive assessment of the acceptability and feasibility of the different modalities of follow-up from patients’ perspectives.”*

- (2) Describe the design in terms of the purpose, priority and sequence of methods: In addition to what has been added to point (1) above, we have added that the mixed-method study was conducted *“following a convergent parallel design”* (l. 139)
- (3) Describe each method in terms of sampling, data collection and analysis: All information regarding the methodology of both quantitative and qualitative sampling and data collection and analysis has been provided in the manuscript (l.151-156 for sampling; l.177-204 for data collection; l.207-231 for data analysis).
- (4) Describe where integration has occurred: We collected and first analysed quantitative and qualitative separately, as it is often done with convergent parallel mixed-methods designs. We then merged the findings (i.e. “integration of findings”) for further analysis and triangulation using the “framework analysis” method. We presented resulted in an integrated way. We have clarified this in the manuscript: *“Both quantitative and qualitative ~~results~~ findings were then ~~merged~~ integrated for joint analysis and triangulation of all results.”* (l.219-220)
- (5) Describe any limitation of one method associated with the present of the other method: We have highlighted several limitations in the Discussion section, for both of the component (e.g regarding profile of participants, study tools, data collection, analysis) (l. 585-627). We do not have any limitation to report regarding our mixed-method approach.
- (6) Describe any insights gained from mixing or integrating methods: Both quantitative and qualitative data showed converging results, highlighting the robustness of the results, as added in the Discussion section: *“The findings showed a convergence between qualitative and quantitative data, thus contributing to the validity of our results.” (*l. 575-577)

Follow below the review comments point-by-point to the authors for each section of the manuscript.

Title
I suggest to the author rewrite the title according to PLOS Global Public Health´s submission guidelines.

**Response.** We have edited the two titles as required:

- Long title
  - Previous title: *“Acceptability and feasibility of home-based and hospital-based follow-up in a COVID-19 clinical trial in Burkina Faso and Guinea: a mixed-method study among patients within the ANTICOV ANRS COV33 Coverage-Africa trial”*
  - New title (now 171 characters, including spaces): *“Acceptability and feasibility of home and hospital follow-up in Burkina Faso and Guinea: a mixed-method study among patients of the COVID-19 Coverage-Africa clinical trial”*
- Short title
  - Previous title: *“Patient acceptability of home and hospital follow-up in a COVID-19 trial in West Africa”*
  - New title (now 69 characters, including spaces): *“Patient acceptability of follow-up in a COVID-19 trial in West Africa”*

Abstract
The abstract was well reported.

Introduction
There are two aims in the last paragraph of the introduction. I suggest to the authors to rewrite this paragraph synthetize the main aim of the study. Because it is not clear what was the aim of the study. The authors in the manuscript body reported other aims. Please, clarify it.

**Response.** We thank the reviewer for their important comment. Indeed, the main aim of the manuscript is to explore the acceptability and feasibility of home-based and hospital-based follow-up in the context of the Coverage-Africa trial, from patients’ perspectives. We have thus deleted the last sentence of the Introduction (*~~We specifically aimed at describing and understanding their experiences and perceptions of being followed up at home or in a hospital~~*) and have edited the objective as follows: *“We sought to explore the acceptability and feasibility of ~~different modalities of~~ home-based and hospital-based follow-up among patients with mild to moderate symptoms enrolled in a COVID-19 clinical trial in Burkina Faso and Guinea.”* (l.102-105)

Materials and Methods

Replace the word “Methods” to “Materials and Methods” according to PLOS Global Public Health’s submission guidelines.

**Response.** This has been edited as required (l.107)

Why did the authors decide to a mixed methods design?

**Response.** As stated in our response above concerning item (1) of the GRAMMS criteria, we used a mixed-methods approach in order to provide a comprehensive assessment of the experiences and perceptions of the study participants. We used a mixed-methods approach in order to provide a comprehensive assessment of the experiences and perceptions of the study participants. The quantitative data aimed to measure in a standardised way the level of participants’ satisfaction and agreement regarding specific statements in a way that would facilitate comparisons between the different follow-up models and between countries. We also originally planned to identify factors (e.g., type of housing, level of education) associated with patients’ satisfaction, which was not possible because of the lack of variability in responses and the very low % of participants dissatisfied. Qualitative data aimed to explore more deeply participants’ individual experiences, views and feelings, and therefore enrich the quantitative findings with subjective and contextual factors.

This is a mixed methods study design. I suggest the authors to organize the headings properly.

Please, organize the level 2 heading in the methods section. Include “Qualitative phase” and “Quantitative phase”. Create the “Data integration” heading.

**Response.** We thank the reviewer for their comment. There are several methodological aspects in common between the quantitative and the qualitative components of our study which include the fact that all data was collected by phone, interviewers were independent from the medical team, study tools were translated in a similar way. We thus preferred to organize the Materials and Methods section without completely separating the quantitative and the qualitative components to avoid repetitions. Nevertheless, for each subsection, we distinguished what was specific to each type of method as follows: “For the quantitative component” (l. 180; l.211) and “For the qualitative component” (l.190; l.213).

Also, as the information regarding “data integration” is presented in the “Data analysis” paragraph (l.219-220), we would prefer not to add a “Data integration” subheading. We hope that based on this rationale, the organization of our manuscript will be deemed appropriate.

Where did the integration of the data happened? The authors stated it happened in the interpretation of the results. It is not clear in the methods section how it happened. It is very important to the reader to understand when the integration of the data and for what it occurred.

- **Response.** Both quantitative and qualitative data were collected within the same timeframe, and by the same interviewers. We first analysed quantitative and qualitative separately, as it is often done with convergent parallel mixed-methods designs. We then merged the findings (i.e. “integration of findings”) for further analysis and triangulation using the “framework analysis” method. We presented resulted in an integrated way. By building on the specificities and strengths of each type of data and then analyzing that data holistically, our goal was to provide a comprehensive and in-depth assessment of patients' experiences, views, and feelings. We have clarified this in the manuscript: *“Both quantitative and qualitative ~~results~~ findings were then ~~merged~~ integrated for joint analysis and triangulation of all results.”* (l.219-220)

In the data analysis heading, please insert a reference about “classical thematic analysis”.

**Response.** We have added the following reference**:** Green, J, Thorogood, N. Qualitative methods for health research (3rd ed.). SAGE Publications Ltd. 2014. (ref 28, l. 213)

I suggest to the authors to clarify the meaning of cultural brokers.

**Response.** Cultural brokers are typically people who are knowledgeable about a specific study setting and local/national sociocultural specificities, and can act both as i) “translators” or “mediators” between researchers from different countries (Guinea, Burkina Faso, France) to ensure that data is well understood by all stakeholders, and also as ii) “validators” to ensure findings are well interpreted, in line with cultural meanings. We have added the following clarification: “*Results and interpretations were validated by the co-researchers in Burkina Faso and Guinea, acting as “cultural brokers” (i.e. providing validation of cultural meanings).*” (l. 229-231). We have also added the following reference: Geertz C. The Javanese Kijaji: The Changing Role of a Cultural Broker. Comp Stud Soc Hist. 1960;2(2):228–49. (ref 31, l. 231)

Results
The results were well reported but I suggest to the authors a better integration of the data. The authors stated in the methods section, data analysis heading “Both quantitative and qualitative results were then merged for interpretation”, but it is nor clear where it happened in the results section.

The results are reported as single studies.

The results reported are relevant but it is necessary to integrate the data to follow the methodologic procedures of the mixed methods study design.

**Response.** Thank you for this comment. We have built on the specificities and strengths of each type of data (quantitative and qualitative) to provide a comprehensive and in-depth assessment of patients' experiences, views, and feelings. The integration of findings showed a convergence between both types of data. However, we realised that specific “cues” could help readers better identify when findings were first issued from quantitative and then qualitative data, or vice-versa. We edited the Results section to better highlight these specificities and types of data integration: see modifications highlighted in blue in the revised manuscript with tracked changes.

As an illustration of what we explained above, we show below 2 different examples of how our mixed-method results section was written.

1) We sometimes first presented quantitative findings, which were the illustrated or nuanced by qualitative data. For example:

- “*Only a few respondents (12%) were worried about not being able to see a healthcare professional every day during their home follow-up (Figure 2); for example, in his interview, one participant from Burkina Faso was convinced that his level of illness would have required hospitalization and close monitoring*.” (l. 286-289)
- *“Moreover, about 25% of participants in Burkina Faso and 45% in Guinea reported being concerned that their neighbors would know they were sick from COVID-19 when followed at home (Figure 2): in the qualitative interviews, participants explained that home visits by medical teams usually trigger curiosity from neighbors – even gossip or indiscretions –, especially when living in a shared compound*.” (l. 300-304).

2) Conversely, we sometimes first presented the qualitative findings, which were confirmed with the quantitative results. For example:

- “*In both countries, participants expressed a sense of reassurance that, in a hospital, there would be increased monitoring and quicker intervention by health professionals if their health deteriorated; in the questionnaires, all the Guinean participants (100%) declared that seeing health care professionals every day was reassuring.*” (l. 354-357)
- “*In the interviews, hospitalization was often perceived as a good strategy to prevent contamination of other family members; this was also reported by 37/38 of questionnaire respondents in Guinea (Figure 2).*” (l. 375-377).

Discussion
I suggest to the authors a deep literature review about the theme. The authors should discuss the results based in studies in different scenarios.

**Response.** We agree with the reviewer’s comments and aimed to support our results with a larger body of literature. However, despite our extensive bibliographic search on PubMed and Scopus, data from the literature was very scarce on our research themes, i.e. the acceptability and feasibility of the different modalities of follow-up. The research equations we have tested were:

- for home-based follow-up: (home[Title]) AND (percept*[Title] OR experienc*[Title] OR accept*[Title] feasib*[Title] OR view*[Title] OR willing*[Title] OR motivat*[Title] OR attitude*[Title] OR perspective*[Title])
- for follow-up of non-severe patients in a health centre: (hospital*[Title] OR confine*[Title] OR facilit*[Title] OR centre*[Title] OR isolate*[Title] OR inpatient[Title]) AND (percept*[Title] OR experienc*[Title] OR accept*[Title] feasib*[Title] OR view*[Title] OR willing*[Title] OR motivat*[Title] OR attitude*[Title] OR perspective*[Title])
- for phone-calls: (phone[Title] OR mobile[Title] OR m-health[Title]) AND (percept*[Title] OR experienc*[Title] OR accept*[Title] feasib*[Title] OR view*[Title] OR willing*[Title] OR motivat*[Title] OR attitude*[Title] OR perspective*[Title])

If the reviewer is aware of interesting papers that we have missed, we would be interested to read them, and where relevant, refer to them in our Discussion.

I suggest to the authors a discussion based in the data from two different paradigms, quantitative and qualitative and how the mixed methods design' findings can subsidize the policy makers in different scenarios to obtain the comprehensiveness of the acceptability and feasibility of home-based and hospital-bases follow-up in a COVID-19 context.

**Response.** The qualitative data contributed to make quantitative results more understandable, providing contextualisation and more in-depth understanding of mechanisms at stake. The quantitative data helped to provide an overview of the magnitude of qualitative findings. The combination of both data sources, showing convergence between quantitative and qualitative findings regarding acceptability and perceived feasibility with the different follow-up models implemented during the trial, contributes to increase the validity of the data. In addition, each method allowed to highlight nuances that are important to consider when planning the implementation of a trial, or developing any public health strategy; we believe this contributes to inform policy-makers to make decisions about the best scenarios to implement in their context.

Are there other limitations? For example, in the individual interviews or in the statistical analysis or in the theoretical framework, etc.

**Response.** In addition to the limitations we have already stated regarding the population and sample (definition and size), the development of the questionnaire in a short period of time, the fact that we did not explore the issues pertaining to the outpatient setting, the fact that data were collected by phone, and the risk of desirability bias, we have added the following limitations:

- Regarding statistical analysis, we have added: *“Moreover, the questionnaire was drafted in a very short period of time, in a context of epidemic emergency where the research protocol and tools had to be quickly submitted for ethical approval; some questions may have been too simplistic. This may explain the lack of diversity and contrast in some responses, and consequently that we were unable to perform statistical tests to determine whether certain socio-demographic factors were associated with participants’ perceptions.”* (l.606-611)
- Regarding the theoretical framework, we have added: “*Also, we have used Sekhon’s Theoretical Framework of Acceptability only at the analysis phase and not us it specifically for the development of the questionnaire and the interview guide: this may explain why we did not find results for all constructs of acceptability (as shown in the S1 Table)*”. (l.613-616)

Conclusions
The conclusions section was well reported.

I suggest to the authors to insert in the conclusion section a little bit about the impact of the patients’ experiences and perspectives in planning clinical trials, considering the COVID-19 pandemic and the necessity of thinking about the participants in clinical trials as people full of experiences, fears and yearnings. According the results of the study.

**Response.** Thank you for this comment. We have added the following sentence in the Conclusion section: “*The results of this study highlighted the importance to conduct formative research on patients’ perspectives, concerns and needs according to their living contexts, when planning clinical trials, especially in the context of an epidemic emergency*.” (l.643-645)

References
Replace the word “bibliography” to “References” according to PLOS Global Public Health’s submission guidelines.

**Response.** This has been edited as required (l.660)

Please, insert the DOI number in the published articles when it is available.

**Response.** We have now added the DOI number for published articles.

Figures
The figures were well reported and provided a better overview about the study, principally figure number 3.

**Reviewer #2:**

Plazy and colleagues conducted a mixed-methods study to evaluate the acceptability and feasibility of follow-up strategies of patients with non-severe COVID-19 recruited in the ANTICOV ANRS COV33 Coverage-Africa trial. Their main conclusions are that hospital-based follow-up was viewed as a way to prevent contamination of family members, but could be badly experienced when mandatory and conflicting with family responsibilities and commitments. Phone calls were seen as reassuring and as a way to ensure continuity of care.

The article is well-written, methods are sound, and the results are presented and discussed overall in a proper manner. Major limitations are adequately acknowledged.

I only have a major concern, which refers to the overlap/confusion of follow-up strategies as per guidelines vs. per trial procedures and how this is reflected in the text. In particular for patients treated in Guinea, all of whom were hospitalized in the initial steps of the infection, it is unclear how the interaction with healthcare professionals not directly involved in the trial might have had influenced their perspective. Could you please elaborate on this?

**Response.** We thank the reviewer for their important comment. Indeed, Guinean patients who were hospitalized could have interacted with healthcare professionals not directly involved in the trial, and we agree with the reviewers that this may have influenced their experiences, particularly knowing that patients mostly reported views about the place where they were followed-up rather than about the follow-up by healthcare professionals involved in the trial team per se. We have thus added the following sentence in the limitations subsection: “*In addition, patients mostly reported views about the place where they were followed-up rather than their views about the follow-up by healthcare professionals per se: this may have led to some confusion, especially in Guinea where all patients were hospitalized at the onset of their infection, as interactions with healthcare professionals who were not directly involved in the trial might have influenced their perspectives and experiences of the trial follow-up*” (l.621-626)

As minor comments:

1. In my opinion the abstract does not reflect all the major findings. For instance, home-based follow-up is not clearly addressed. Or "Our results also highlight the importance of emotions in the acceptability of trial follow-up models and suggest that emotion-based considerations should be included when assessing the feasibility and planning the
implementation of a trial, or developing any public health strategy."

**Response.** Thank you for this suggestion. We have edited the last sentence of the Abstract: “*These overall positive findings support the development of home-based follow-up for mildly ill patients in West-Africa, ~~considering both~~ provided that both emotional and cognitive factors at individual, familial/inter-relational, healthcare and national ~~level~~ levels* *be addressed when planning the implementation of a trial, or developing any public health strategy*.” (l.52-56)

2. Methods, study setting. Please, include a clear definition +/- reference for "non-severe symptoms form of the disease"

**Response.** Non-severe forms of the disease were defined as having peripheral oxygen saturation above or equal to 94% measured by pulse oximetry. We modified the methods section for clarification as follows: “*Patients eligible for trial enrolment were at an early stage of their disease (less than 7 days of symptoms), had symptomatic but non-severe disease, i.e. with peripheral oxygen saturation equal of above 94% as measured by pulse oximetry, and were 40 years of age or older, or 18 years of age or older with comorbidity including obesity, treated hypertension or treated diabetes mellitus.*” (l.116-120)

3. "Trial procedures regarding patient enrolment and follow-up were defined in accordance with the COVID-19 national recommendations prevailing during the study." This sentence deserves further detail, in particular regarding whether this might have affected the profile of patients enroled and this, in turn, has affected the follow-up procedures and therefore how participants in the study have perceived them.

**Response.** In Burkina Faso, patients with COVID-19 were allowed to stay at home per national recommendations; since the proposed follow-up in the study was feasible at home, we feel that this did not affect the profile of the patients enrolled in the study. In Guinea, as all COVID-19 cases, whether symptomatic or not, had to be hospitalized, we cannot exclude that those paucisymptomatic may have chosen not to be tested and hospitalized, hence this could have prevented some more reluctant individuals to be invited to participate in the Coverage Africa trial. We have added this in the limitations: *“Yet, we acknowledge some limitations regarding this study. We included only individuals who already consented to participate in the trial, i.e. de facto had accepted the models of follow-up proposed in the trial (aligned to that of the country). In Guinea, national recommendations at the time of the trial - i.e. all individuals who tested positive for COVID-19 had to be hospitalized - may have caused some people to avoid getting tested [36]: this could potentially affect the profile of the patients enrolled in the trial if the latter had different characteristics compared to the patients included. Also, one of the documented reasons for refusal to participate in the Coverage-Africa trial was the fear of a high number of follow-up visits and calls […].”* (l. 585-592)

Regarding trial follow-up procedures, they were actually developed in accordance with the national recommendations. We have clarified the Materials and Methods section, specifying that the national management recommendations are the ones explained above the paragraph: “*Trial procedures regarding patient enrolment and follow-up were defined in line with these national COVID-19 management recommendations prevailing during the study period*” (l. 130-131).

4. In the limitations, authors already tackle the potential selection bias by level of education. However, could you please elaborate on the fact that only two patients leaved alone (beside the community-oriented society aspect) and how might have impacted the findings and the potential external validity?

**Response.** In our study sample, two participants from Burkina Faso reported to live alone which corresponds to 1% of our study population in Burkina Faso. This is line with the last census of Burkina Faso (2022) showing that very few households were composed of only one person.

<http://www.insd.bf/contenu/documents_rgph5/VOLUME%202_CARACTERISTIQUES%20DES%20MENAGES%20ET%20DE%20LA%20POPULATION%205E%20RGPH.pdf>

We agree however with the reviewer, and this is stated in the limitations’ sections, that the overall sociodemographic characteristics of our study population (level of education and housing conditions) may indicate a selection bias, thus may reduce the external validity of our findings.

Regarding the educational/socioeconomic position, could the bias prevent identifying structural barriers for at home treatment and follow-up such as individual rooms, open air spaces, availability of mobile phones, etc? Could you please elaborate on the relationship between socioeconomic position and fear of stigmatization.

**Response.** We agree with the reviewer that the profile of our study population may have reduced the likelihood of uncovering structural constraints in the home follow-up experience. We mentioned this issue surrounding stigmatization and socioeconomic position in the Discussion section: “*Many participants had a “privileged” profile, i.e. living in a home with a private yard or having a relatively high level of education, which may explain why few of them reported fearing stigmatization*” (l.594-596). We added the following sentence, as suggested by the reviewer: “*this potential socioeconomic bias related to participant selection may also have prevented the identification of structural barriers regarding home and phone-based follow-up, such as access to individual rooms*.” (l.596-598).

5. Any particular conclusion from the interviews of those patients that received hospitalization only (71% of patients from Guinea)? Is shocking that one of the highlighted statements is: "However, 50% of participants from Guinea shared that it was difficult to be hospitalized for COVID-19 while they were not having severe symptoms. This was also shared in the individual interviews, as some participants perceived that their health condition did not require hospitalization." All participants had non-severe COVID-19 and were all hospitalized.

**Response.** As highlighted by the reviewer in their statement and as reminded in our answer to comment #3, per national requirements, all COVID-19 patients in Guinea had to be hospitalised, whether they had severe or non-severe symptoms, or were even asymptomatic. However, none of the Coverage-Africa study participants were hospitalized throughout their follow-up: they all were discharged home at some point during their 28-day follow-up.

The “location until D8” variable presented in table 1 shows that 71% of Guinean patients were hospitalized up to the 8^th^ day of follow-up. As stated in the manuscript, most patients who participated in the qualitative interviews were discharged home at some point before the end of their follow-up in the trial (l. 251-253).
